# Contact-Free Multispectral Identity Verification System Using Palm Veins and Deep Neural Network

**DOI:** 10.3390/s20195695

**Published:** 2020-10-06

**Authors:** Maciej Stanuch, Marek Wodzinski, Andrzej Skalski

**Affiliations:** Department of Measurement and Electronics, AGH University of Science and Technology, Al. Mickiewicza 30, 30-059 Krakow, Poland; wodzinski@agh.edu.pl (M.W.); skalski@agh.edu.pl (A.S.)

**Keywords:** biometrics, palm vein scanner, multimodality, convolutional neural networks

## Abstract

Devices and systems secured by biometric factors became a part of our lives because they are convenient, easy to use, reliable, and secure. They use information about unique features of our bodies in order to authenticate a user. It is possible to enhance the security of these devices by adding supplementary modality while keeping the user experience at the same level. Palm vein systems are based on infrared wavelengths used for capturing images of users’ veins. It is both convenient for the user, and it is one of the most secure biometric solutions. The proposed system uses IR and UV wavelengths; the images are then processed by a deep convolutional neural network for extraction of biometric features and authentication of users. We tested the system in a verification scenario that consisted of checking if the images collected from the user contained the same biometric features as those in the database. The True Positive Rate (TPR) achieved by the system when the information from the two modalities were combined was 99.5% by the threshold of acceptance set to the Equal Error Rate (EER).

## 1. Introduction

Biometrics is a technique of authorization and recognition based on many characteristics of a human body and behavior that are unique and that can be used for distinguishing one subject from another. The power of biometrics is hidden in its simplicity and reliability. The user does not have to remember complicated passwords that are advised to be changed from time to time. Users tend to use the same password for many sites or to use ones that are simple so they can memorize them. If a complicated password is required, people often write it down for the sake of convenience and it becomes vulnerable to theft [1]. Biometric authorization methods are superior compared to the aforementioned issues. The user does not have to remember any passwords and will never lose them due to the fact that the authorization key is bound to the particular body [2]. On the other hand, there is a problem related to data storage. There is always a risk of a leak of the database of users’ biometric profiles, and it could pose a big threat to the society as the biometric information cannot be changed since it is bound to our physiology. Thankfully, it is not an issue if the database is created with care. The biometric information should not be stored in a raw form but rather as a set of extracted features with added noise. It should be additionally hashed with a personal key that might be changed in case of a security issue [3]. Another concern worth mentioning is the problem of forging a biometric information using different techniques. There were numerous cases where hackers were able to break into some of the systems thanks to obtaining the biometric information and imitating it accurately [4]. Such a situation happened in 2014 in Germany and was a warning sign to all people developing biometric solutions. The German Defense Minister, Ursula von der Leyen, was hacked by Jan Krissler who used commercial photos of the Minister and used them to replicate her fingerprints [5].

Different biometric authorization and verification techniques can be rated in multiple classes like fraud resistance, easiness of use, time of procedure, or hygiene. One of the most interesting and fraud-resistant biometric methods is based on patterns of blood vessels [6].The images acquisition can be focused on specific body parts like finger vein [7,8], palm vein [6,9], dorsal vein [10,11], or wrist vein [12,13] systems. It turns out that the palm vein pattern is unique for every human, even between twins [14] and can be used in the terms of authorization and recognition systems for the most important systems (banking, military etc.). It is believed that it is more secure than fingerprint systems because we tend to leave fingerprints on almost every surface we touch. Furthurmore, we can also get a full image of the fingerprint from a camera photo [5]. It leads to multiple infringement possibilities for wrongdoers. The palm vein systems do not suffer from this issue as an image of the veins is not normally visible in standard lighting conditions [15]. Specific conditions must be satisfied in order to get an adequate image. Thanks to this fact, palm vein systems are rising in popularity around the globe. Unfortunately, there are still ways to hack such systems. In 2018, a live demo of hacking the palm vein system of Fujitsu was presented, again by Jan Krissler, with detailed steps of how to collect images without notice of a person that is a target and he also presented how to create a wax model that can fool the system [16].

Liveness detection is a countermeasure for such attacks and is designed to prevent biometric fraud [17]. The main goal is to stop spoofing attacks. The aim of these means is to check whether the user is a living human presenting their body for a scan or is just a nonliving item that tries to imitate the biometric information and to fool the system as a result. Liveness detection can be achieved in many different ways, e.g., by checking vital signs such as variability of the image caused by blood flow [18]. Another option is to do a spectroscopy scan of the object that is being scanned and to check its similarity to the human tissue spectrogram [19]. Moreover, there are also methods that are focused on the conductivity of a tissue, but they require direct contact with a human body [20].

For the purpose of spoofing the system, one can print images using a commercial printer and create wax models or even 3D-printed models.

In [21], the authors proved that a palm vein system without a liveness detection system can lead even to 65% of false acceptance rate. In order to overcome these limitations, we propose a system that uses not only Near Infrared (NIR) but also ultraviolet (UV) lighting as a defense against spoofing attacks. The short waves of UV expose the fine details of a hand while hiding information about veins that are not visible in this illumination [15]. This makes it possible to acquire the palm print [22] image that carries additional biometric information. Comparing NIR and UV images makes presentation attacks unlikely to be successful. Therefore taking images using NIR and UV illumination in a matter of seconds is a desired feature. If these two steps were separated, the impostor would have many more occasions for an attack. It is an example of a multimodal biometric system which is characterized by a greater recognition efficiency, greater security, and greater reliability than a classic unimodal biometric system [23]. UV light is divided into 3 groups based on wavelength: UVA (400–320 nm), UVB (320–280 nm), and UVC (280–100 nm). From the perspective of biometric systems, UVA, which is the main part of the UV radiation that comes with sunlight, is in thet range of interest. This might be carcinogenic to skin as it causes oxidative damage in skin cells [24]. Therefore, it is crucial to check the potential effect of the system on skin. Thanks to very low radiation and short exposure times, it does not pose a direct threat, which is described thoroughly in the Hardware Section 2.2.

### Related Works

Some authors proposed contactless biometric systems that use the IR spectrum. In [25], such a system was proposed. The multimodality comes from the information about vein patterns and hand geometry. A complicated lighting system was used to obtain the images. Biometric features were extracted by using adaptive thresholding, median filtering, and skeletization.

Another multimodal biometric palm vein system was proposed in [26]. The second and third modalities in this work were fingerprint reading and face recognition. Both were gathered using additional devices. It is a drawback for the user as it means that more action is needed in order to be authorized. The biometric feature extraction was done using traditional approaches for every one of the aforementioned modalities (Scale-Invariant Feature Transform (SIFT) features for the palm vein). GoogLeNet convolutional neural network was fine-tuned on databases and was used in order to detect spoof attacks.

The system described in [27] is single-modal and checks only palm vein features. The system is contactless and uses multiple Gabor filters for biometric features extraction.

In [28], the system was not described but the researchers gathered a database for a contactless palm vein system where they scanned 103 people and got 1260 samples. The work focused on gaining biometric features in a rotation invariant manner. It is crucial for systems without a hand support.

A multimodal approach with Convolutional Neural Networks was used in [29], where the first modality is the fingervein image and the second is the finger shape. It was done with a single sensor so it does not influence the user experience.

In [30], two different images, a palm print and a palm vein one, were fused together in order to achieve better results while preserving protection against possible fraud. The system described consists of an IR camera and a low-resolution digital scanner. The two images were fused in order to combine the biometric information, resulting in an accuracy of 98.8% with a False Rejection Rate (FRR) of 1.2% and a False Acceptance Rate (FAR) of 1.56%. The authors gathered a database that consisted of 100 palms and 6000 images.

## 2. Materials and Methods

We have constructed a multimodal biometric system which uses palm images that are taken in the UV and NIR ranges during the same examination which is a unique feature. The system is contactless, provides the result in a manner of milliseconds, and can be used in a variety of biometric applications where user validation is needed. A database of images was collected in order to check the performance of the system. In this work, we focus on feature extraction techniques using convolutional neural networks for both IR and UV palm images without a very specific preprocessing that would extract the features in a deterministic way like it is done, for instance, using vein filters.

The previous work on this system was focused on a deterministic approach using Local Binary Patterns (LBP) as an element for feature extraction along with feature parametrization based on an elastic deformation. As a result, the TPR of 97.69% was achieved [31]. Since then, the database was expanded greatly, and now, it is more appropriate to fit the neural network methods, which seems a reasonable solution to the biometric verification and recognition problems.

### 2.1. Overview

In this section, the following topics are covered. Firstly, hardware setup is discussed. Then, the structure of the database and the enrolment process is described. After that, the neural network architecture that is used for the purpose of the feature extraction is presented. In the next section, the method of image preprocessing can be found. Subsequently, the learning process and metrics are described. In the last subsection, how the modalities are combined in the system and how they can be used to provide liveness detection feature are explained.

**Figure 1:**
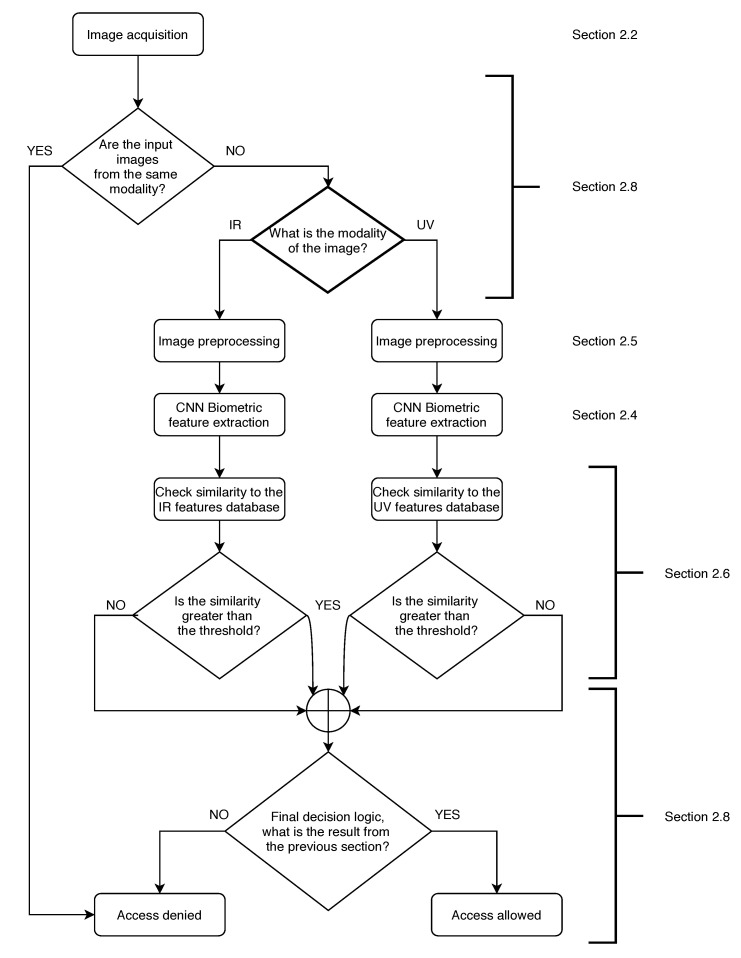
Block diagram with an overview of the system workflow that represents the verification scenario where we minimize false negatives.

The overview of the system workflow is presented in Figure 1. User verification starts with image acquisition; then, it is checked whether the input images are from the same modality. If they are, access is denied as the user is an impostor. If not, the input image is directed to the IR or UV pipeline of image preprocessing, feature extraction, and similarity checked with the database. Afterwards, it is checked whether the similarity to the database is greater than the threshold that is set after the training process. The next step is to compare the results from the IR and UV pipeline as described in Section 2.8 and based on decision logic. Finally, the result of the verification is presented.

### 2.2. Hardware

**Figure 2:**
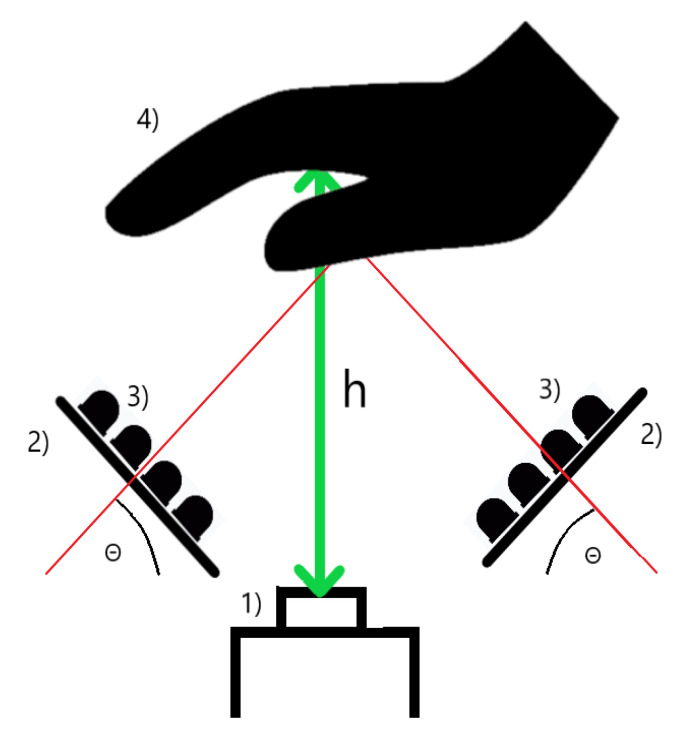
Overview of the system, where (1) is the Charge Coupled Device (CCD) sensor, (2) is the illuminators, (3) is a mix of the NIR and UV diodes, (4) is the hand of the user, (θ) is the 45 degrees angle, and (h) is the distance between the users’ hand and the sensor [32].

We constructed a system that consists of a Charge Coupled Device (CCD) camera that has a good sensitivity in both the NIR and UV ranges (JAI CM-140-GE, a relative spectral response of the camera is nonlinear and, around the UV range, it is in the compartment of <0.45,0.65> of the maximum response; 0.45 is the response for the 390-nm wavelength and 0.65 corresponds to the 450 nm wavelength. In NIR illumination, the relative response starts from 0.4 at the 750-nm wavelength and ends at 0.25 of the maximum response at 900 nm) and two lighting panels that are of our own design. The sensor is also equipped with a polarization filter. The panels (illuminators) consist of light-emitting diodes (LED) that emit light in the NIR and UV wavelengths ranges and a diffuser that diffuses and scatters the light. The diodes are set uniformly at an angle of 45 degrees in order to obtain a homogeneous illumination, and the hand of the user should be 30 cm (with the acceptance range of +/− 5 cm resulting in a <25;35> cm range) over the sensor. There is a tripod with an indicator that suggests the correct height over the sensor. There were two diode types used in the NIR range. Half of them have the peak at 850 nm, and the other half have the peak at 940 nm [32]. This approach gives the combined information that can be gathered in the aforementioned range. These wavelengths correspond to the optical window for in vivo imaging that allows for subcutaneous vein imaging [33]. The UV diodes have a peak at 395 nm. The diodes used have a typical luminous intensity of 20 millicandelas, which translates to 0.00757 lumens, for the diodes used, which can be converted to 0.0841 milliwatts. There are 20 diodes used, and that means they produce around 1682 milliwats. An average human hand has an area of 0.014 m${}^{2}$ [34]. Having this information, we can calculate that, without a diffuser, we would illuminate the hand with 120 mW/m${}^{2}$ for around a second. Based on information from [35], we can calculate the UV index for the system by dividing 120 mW/m${}^{2}$ by 25 mW/m${}^{2}$. This gives a UV index of 5, which corresponds to a moderate risk of skin burn during a long exposure [36]. Thanks to the very short time of the scan, the UV radiation produced by the system does not influence overall human UV exposure by any significant amount that would pose a health threat. The images were originally captured with the resolution of 1392 × 1040. There is no object like glass or a handle that would be in touch with the hand that is scanned. The user can hover the hand freely over the scanner without any element that would hinder the movement. Figure 2 is an accurate depiction of how the system is built. This approach provides the possibility to scan the hand at two different wavelength ranges (UV and NIR) in a matter of milliseconds, which prevents fraud in the biometric systems. The images are taken in a randomized order so the users do not know whether the NIR or UV scan comes first in the process, which makes presentation attacks more difficult as the impostor would have to guess the correct order of the process or would have to imitate the changes in the images that normally occur when different lighting is employed. Furthermore, it is possible to repeat this process multiple times during the verification procedure [37].

### 2.3. Database

**Figure 3:**
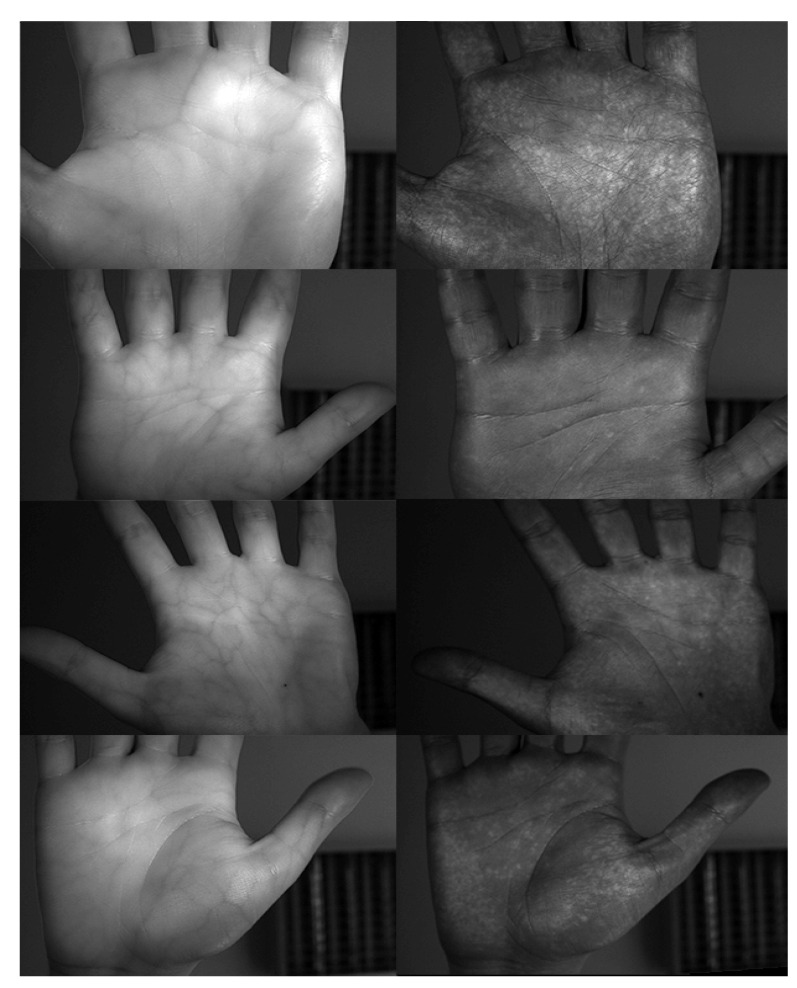
Example of images taken in IR (**left**) and UV (**right**) lighting from the database.

In order to check the performance of the proposed system, there was a need to acquire a database. We were able to collect pictures from 515 different persons using the proposed system. For every hand of every person in every lighting condition, there were 8 images captured. For every modality, the person hovered their hand over the scanner and moved it back. The hovering took approximately one second for a single approach.This was done 4 times for every hand. Every time the hand hovered over the scanner, two images were taken. Sample images are depicted in Figure 3.

A clean up of the database was needed, as it turned out that some of the images were not sharp enough in order to contain reliable biometric information. These could not be taken for further testing, but there were too many scans in order to assess every image manually. That is why we used an automatic approach to check image blurriness. A variance of Laplacian was used as a metric for detecting whether an image was sharp or not. The Laplacian *L(x,y)* of an image with pixel intensity values *I(x,y)* is given by

(1) $$L\left(x,y\right)=\frac{\delta^{2}I\left(x,y\right)}{\delta x^{2}}+\frac{\delta^{2}I\left(x,y\right)}{\delta y^{2}}$$

In cases where the edges were sharp, the variance was high, and low variance means that the image is blurred. The sharper the image, the better the quality of a biometric feature. That is why, for future processing, five of the sharpest images of every person’s left and right hands were taken for further training and testing of the neural network.

It is worth mentioning that, in real-life scenarios, the sharpness of an image is not an important issue. When a blurred image is detected, another picture can be taken which resolves the issue. Almost 30 shots might be taken during one second so it gives a lot of possibilities in that matter.

Overall, after the cleanup, the database consisted of 10,160 images; 5080 taken in NIR lighting and 5080 taken in UV lightning. The images of right and left hands are considered scans of different individuals. This results in 1030 subjects that are used for training of a neural network.

The database was divided randomly into 3 groups with a uniform distribution of left and right hands between groups. Three images of a single hand from every examination (overall 1030 subjects, 3090 NIR and 3090 UV images as pictures of left and right hands are considered different subjects) were submitted to the training group, one to the validation group and one to the test group. As a result, the images were divided as presented in the Table 1:

The 3 images of one subject used for the training group resembles the enrolment process which is done while the user registers. The validation group was used to check the neural network performance during the training process, and the test group was used to check the overall performance of the model.

### 2.4. Neutral Network Architecture

**Figure 4:**
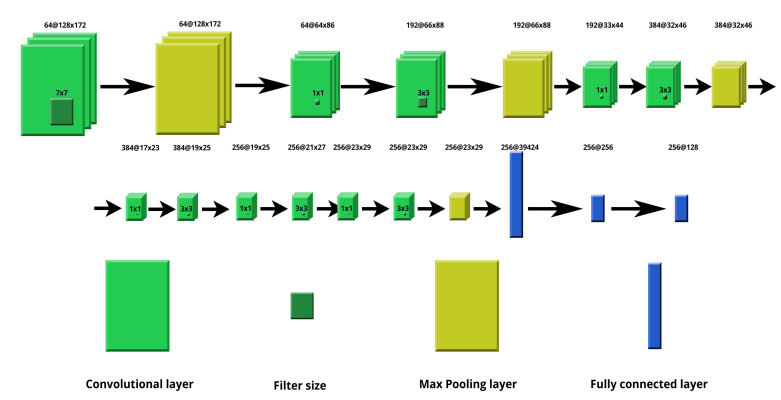
Neural network architecture.

We decided to use a neural network approach for the purpose of feature extraction. This method provides a great flexibility; it does not require a complicated image preprocessing step. The neural network architecture chosen was a convolutional neural network (CNN) with convolutional, max pooling, and fully connected layers. They were primarily used for object recognition in image processing. This solution is able to detect important features without any human supervision as the layers function as filters that are refined during the learning process. The layers create feature maps of image regions that later are broken into rectangles and sent out for nonlinear processing. Max pooling layers allow for downsampling an input representation. Max filter takes the maximum value of a feature from nonoverlapping regions in the process. It is possible to use different filters like an average filter. It helps in preventing overfitting, and it also reduces the computational cost. The fully connected layers take inputs from feature analysis of the previous layers, reorganize them into a single vector, and apply weights to it. As a result, a feature vector that can be used later is produced. This architecture is computationally efficient and can be used in many fields as there is no need for extracting specific features manually. An important drawback of the method is that lots of training data is required. Parametric Rectified Linear Unit (PReLU) was used as a activation function. The activation function is crucial in deep learning because it influences the learning process by changing the computational efficiency of training a model. PReLU is used because it resolves the vanishing gradient problem of sigmoid activation functions [38]. The proposed architecture is composed of 10 different layers: 5 convolutional, 4 max pooling, and 1 dense layer. The same architecture was used for the IR and UV images. The architecture used is presented in Figure 4.

### 2.5. Image Preprocessing

Only a straightforward preprocessing was performed in order to simplify the solution in contrast with deterministic approaches where this step is crucial and must be done with care.

The resolution of every image was reduced to 348 × 260. This reduction is needed to comply with the proposed CNN architecture. A different size would require a change of the CNN as there is a need to preserve the receptive field of the network [39]. The size of an image is always a trade-off between the computational efficiency and the accuracy as lowering the resolution increases the possibility for many more epochs and bigger batch sizes during the training process. Lowering the image size makes the whole process faster, but the effect on the accuracy of classification is often significantly lower [40,41]. The images are monochrome as they consist of one channel of data. The training and validation group was augmented thanks to simple transformations such as cropping and resizing in the factor range of 0.8 to 1.2 of the reduced image size. The images from the test group were not augmented. This process was crucial for efficiency of the training of the neural network. Without augmentation, the neural network was not generalizing knowledge properly. No Region of Interest (ROI) was extracted in order to feed the neural network as we wanted to keep the whole process as simple as possible.

### 2.6. Learning Process

The first thing to do after the database acquisition and preprocessing step was training of the neural network. The learning process was carried out using triplet loss function [42], which is done as follows: there are two pictures chosen out of three possible images for the same person and one out of three possible pictures chosen for another person. The three images are shown to the neural network, and then, we get a descriptor for the three images. Euclidean distance between these sets of features is checked. If the result is higher than a threshold of 0.4, it is assumed that the presented images represent biometric features of the same person. This was determined automatically during the learning process. By the chosen threshold, the EER had the lowest value. The same threshold was employed for all cases. The learning parameters were as follows: 500 epochs, learning rate of 0.0001, momentum of 0.9, batch size equal to 16, and margin equal to 1.0. The same optimization parameters were used as for the NIR and UV modality images.

### 2.7. Metrics

In order to evaluate the results an equal error rate (EER) was checked for all of the given cases. EER is a point where the true positive rate (TPR) equals the true negative rate (TNR). This approach gives a good overview of the strength of a classifier in biometric systems as it provides a comparable and reproducible compromise between acceptance and rejection rates. EER was calculated by plotting the results over a range of thresholds.

(2) $$TPR=\frac{TruePositive}{Positive}$$

(3) $$FPR=\frac{TrueNegative}{Negative}$$

### 2.8. Comparing Modalities

The system is based upon the capability of capturing images in two different modalities. At first, the images from NIR and UV have to be checked for similarity. They are both taken within a second, and as a result, the motion artifacts between the images are negligibly small. Any visible differences should come only from the different illumination conditions. In the NIR lighting, darker regions resembling the subcutaneous veins should be visible thanks to the optical window for the in vivo imaging that is described in Section 2.2. The UV images are characterized by the darker look of the skin. The veins should not be visible due to the aforementioned optical window, and friction ridges shall be observable (with the exception for people that suffer from Adermatoglyphia who do not develop finger and palm prints [43]). Additionally, little white spots might be visible in UV lighting. There are many uses of the additional scan. Some of them will be presented in this section.

Having the two images, we can check whether they are the same image by comparing them. This is crucial from a security perspective as it makes a personification attack harder. The simplest way of checking whether the scanned objects have differences is to subtract the images. The disparity can be clearly visible and easily noticeable by using Sum of Square Differences or a Cross-Correlation. When the images have no adequate differences, we have a case of a presentation attack and access should be denied.

After checking the similarity of the images, it is important to assign images from the correct modality to the corresponding pipeline (NIR or UV). Even though the sequence of taking images is randomized, it is known to the system. Based on this information, images are distributed to the appropriate pipelines that are also shown on Figure 1.

There are multiple ways of defining the final decision criteria. The most straightforward one is to use only one of the modalities for the purpose of user verification. This approach is the simplest one, but even in this case, the security of the system is enhanced by the first step of the system which is the NIR and UV image comparison. The verification results remain unchanged, but there is the possibility to stop impostors from compromising the system.

Another way of using the additional information is to get the biometric features of NIR and UV images and do a comparison for each modality. The system might allow access only for users with positive results from both illuminations. In this case, we end up lowering the EER and boosting the security of the solution. This might be adequate for solutions that need maximum security where we want to maximize the True Negative Rate (TNR) and to minimize false positives.

The last proposed approach is focused on minimizing false negatives. When the result of one of the comparisons, from NIR or UV lighting, is positive, then the overall result is also positive. This approach gives users more convenience in using the system as the false negative cases are minimized and it lowers the possibility of the need for a second scan when the first one fails. This approach might cause an increase in false positive cases.

The final results were evaluated with the decision logic, which was optimized for minimizing the false negatives. The user is verified when there is a match in the database in at least one of the modalities.

## 3. Results

**Figure 5:**
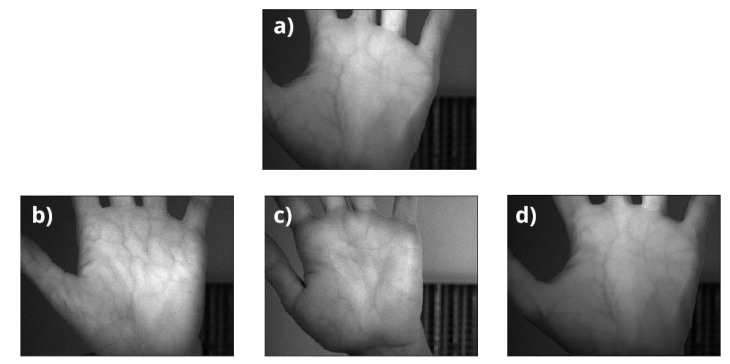
An example of comparison: (**a**) is the image that is captured when the user tries to authenticate itself, (**b**–**d**) are some samples from the database where (**b**,**c**) and are images of different persons and (**d**) represents a match in the database.

An example of the results is presented on Figure 5. The input of the system (a) is processed by the CNN, and we obtain a feature vector as a result, which we can compare later on with the information stored in the database.

**Figure 6:**
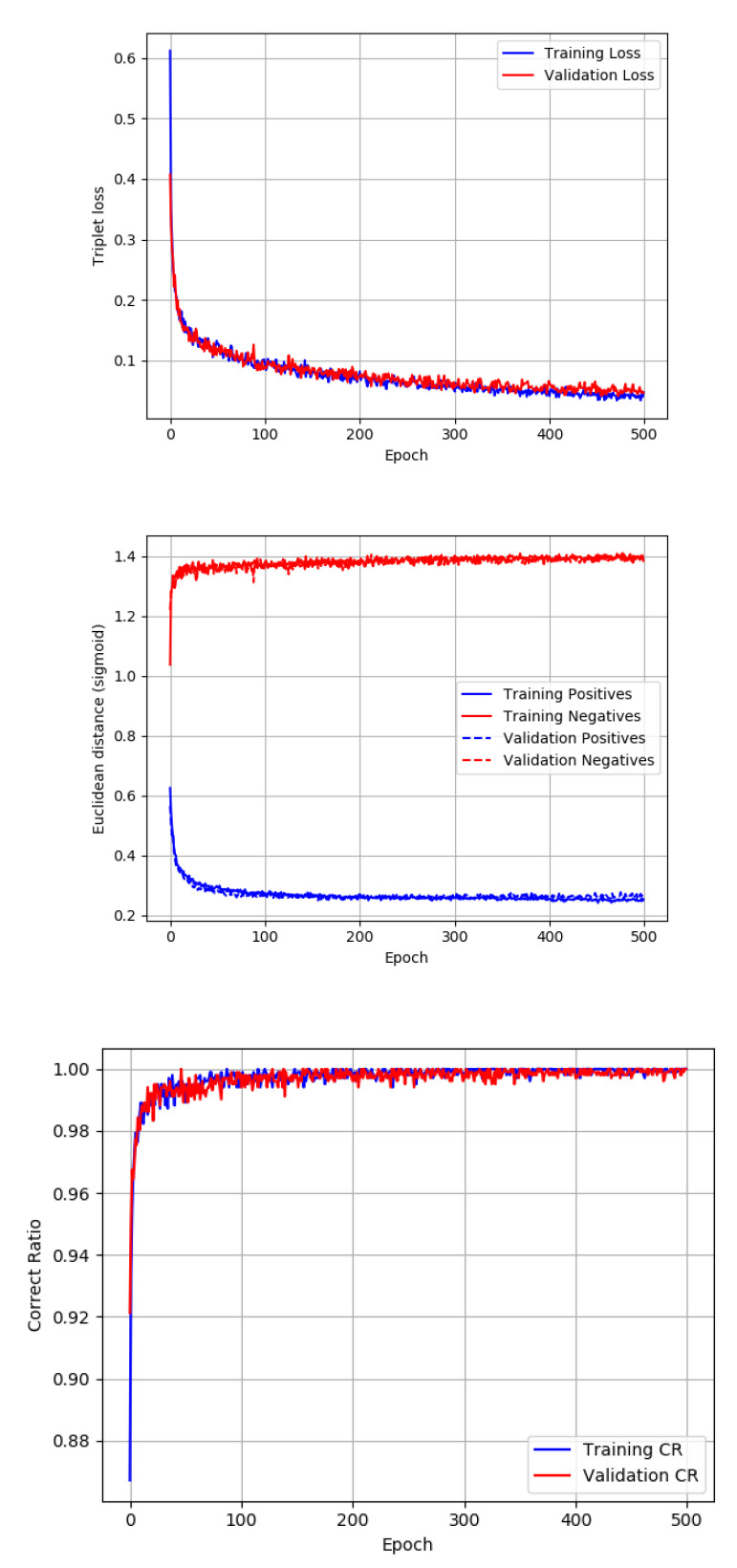
Training process for the IR dataset.

With only very simple image preprocessing, we were able to obtain satisfactory results in both modalities thanks to the proper setup of the system. As it can be seen in the Figure 6 generated during training, the neural network was not overfit on the given 500 epochs on both the IR and UV datasets. A lower number of epochs resulted in a lower TPR, but a higher number of epochs exceeded the time of training significantly while validation loss did not improve. That is why this number of epochs was appropriate for the given task. The batch size was proposed to be 16 as it is a reasonable tradeoff between memory usage and the performance of the network.

The NIR and UV modalities were tested for their accuracy in order to check whether the neural networks learned how to extract biometric features from images taken under two different illuminations. All of the tests in all of the scenarios were checked for the whole range of possible thresholds. Even though changing the threshold can influence the overall results, we standardized the results by choosing thresholds that ensure a balance between error rates, which is the EER.

The next step was to check the performance of the system on the NIR and UV datasets separately. From the plot, it can be learned that the FPR for both NIR and UV datasets are similar but the obvious difference is in the trajectory of the TPR results. The EER of the NIR approach is almost 0.5% better than the EER of the UV approach. It means that the neural network used for feature extraction can distinguish more important details from the NIR images where vein patterns are visible.

The NIR got a TPR of 97.95% and the UV neural network got a TPR of 97.26% by the threshold of the EER. In both cases, it is significantly more than in the previous work where the LBP approach was employed for the task of feature extraction. The previous result that was achieved was on the level between 92% and 96% based on the different approaches while using the LBP [31].

**Figure 7:**
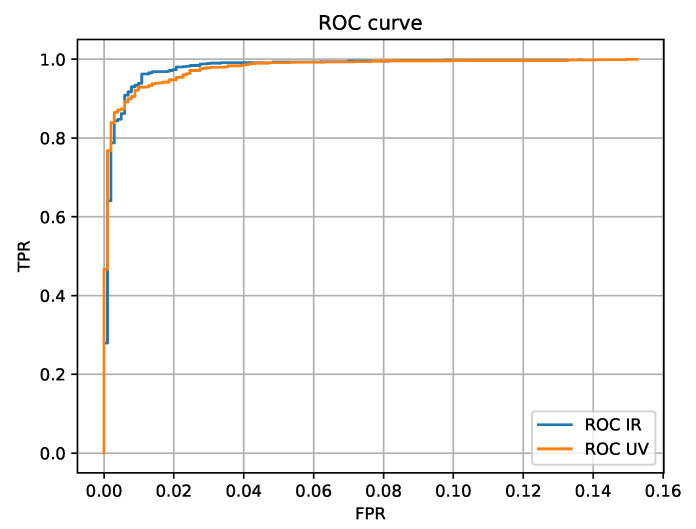
Receiver Operating Characteristic (ROC) curve for IR and UV neural network approaches.

The Receiver Operating Characteristic (ROC) curves for both the IR and UV approaches show significant capability of the model to distinguish between two classes (the same person and another person), which is depicted in Figure 7.

In the case of minimizing false positives with the verification criteria that both similarity checks, in NIR and UV, have to be fulfilled, the outcoming TPR was on the level of 95.9% by the EER threshold, which is lower for UV without NIR, as foreseen. There are cases where the classifier has the proper solution in the UV range and not in the IR.

**Figure 8:**
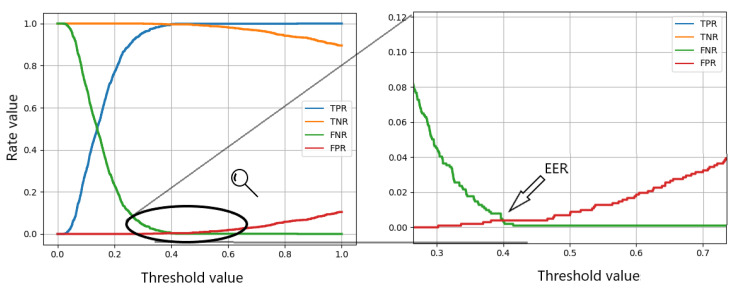
The influence of the threshold value on the True Positive Rate (TPR), True Negative Rate (TNR), False Negative Rate (FNR), and False Positive Rate (FPR) in the scanario of minimizing false positives.

For minimizing false negatives, we can test the solution with the verification criteria that it is enough to pass one of two verification steps in the NIR or in the UV range. The obtained TPR is 99.5% by the EER threshold, which is a much more satisfactory result. Figure 8 presents the results for a whole range of thresholds and shows how they influence the results. It also depicts how the EER is chosen.

## 4. Discussion

A large database is crucial for validation biometric solutions which are expected to work correctly and without mistakes with the whole Earth’s population. Usually, the groups of users are not as diverse and huge, but still, a high level of security is expected. In comparison to the state-of-the-art, we created a significantly bigger database that consists of 10,160 images from 1030 hands (5080 images in NIR). The most popular palm vein database CASIA consists of 100 different people (200 different hands).

It is seen that other methods mentioned in the results section have worse or similar results for the EER, but it is worth mentioning that the database gathered in this work has a greater position variability than the CASIA Palm Vein Database. This was checked by segmenting hands from images using Otsu’s binarization [44]. The centroid position of the biggest of the non-connected elements in the binary image was checked. This was done for all of the images in the CASIA and our database. The position variability was calculated as standard deviation of the X and Y centroid position and was scaled as a percentage of the whole image. The results are presented in Table 2 and indicate that, in our database, the hand centroid position is around two times more diverse than in the CASIA database.

Kang et al. checked the influence of variability of position on an author database, and it proved that it lowers the EER (rows 1 and 2). We have proposed a novel method that was validated on the largest database from all of the known cases, and we were able to maintain low EER while adding position variability. Based on the results from Table 3, we can see that Cancian [27] and Khan [45] proposed methods that performed worse than our solution. Hao [46] and Yan [47] presented solutions that perform similar to our system but with much lower number of images of lesser position variability comparing to the proposed method.

The results prove that the proposed feature extraction method is invariant to the absolute position of the hand in the image. In the preprocessing step, a random crop was used in order to enhance hand position variability during training. This is achieved by the use of filters in the CNN that are not spatially unique. For orientation changes, they are not that vague and the plenitude of the filters is capable of addressing this issue.

The results obtained when only one of the modalities is taken for the verification task are better than in the scenario where false positives are minimized. This indicates that there are different features in NIR and UV images.

The verification results prove that TPR is higher in the NIR range but that the difference was nonsignificant. This is another proof that both modalities can be used for the task of user verification based on their biometrics.

The results of the scenario where the false positives are minimized shows that we can obtain lower TPRs by the EER threshold, which is reasonable as the system does not accept any users of whom it has any doubts. What is more important is the scenario of minimizing false negatives. By verifying the user that passed one of the two tests, the TPR was higher, but it turned out that the EER was also lower. This is important information as we have proved that the use of both modalities can boost not only security by comparing NIR and UV images but also reliability of the system. Additionally, we have more flexibility in adjusting the system for the needs of the industry. Some applications need to minimize false positives and some false negatives.

## 5. Future Challenges

Future works comprise enlarging the database in order to get a more reliable solution for neural network training and the overall validation. It is planned to try different neural network architectures on the obtained database in order to choose the most appropriate network for biometric feature extraction. It is also planned to use images of a higher resolution for training of the neural networks.

The lower result of the UV neural network is most probably caused by the lack of all the fine details that were lost in the process of image downsampling while the resolution was lowered. In future works, it is planned to adjust the image sizes in a way that will preserve biometric information.

Further works should also include experiments with the preprocessing step, which is crucial in the struggle against overfitting. In the experiment, it turned out that the random crop had the greatest impact preventing this adverse phenomena. Nevertheless, proper image augmentation techniques, resizing, and other transformations influence the neural network learning process and that is why performing more tests is necessary in this area. Apart from further plans, it is worth mentioning that the results are better or comparable to the state-of-the-art even though the proposed neural network is relatively simple.

## 6. Conclusions

Palm vein systems are important and valuable for biometric systems though they can still be vulnerable to presentation attacks. Liveness detection is a countermeasure for this drawback, but it is also important to keep the verification process as simple as possible for the user. We proposed a novel biometric system design. It uses the NIR optical window for palm vein imaging and UV illumination for obtaining palm print images. Thanks to this combination, it is possible to obtain more biometric information than in standard palm vein systems. We preserve a high accuracy, and we have an additional layer of security for presentation attacks while keeping the user experience at the same level as no additional action of the user is required. The additional modality gives a lot of new options for adjusting the final device to the user needs. The use of different lighting boosts security and reliability of the system while maintaining the ease of use, which is crucial nowadays. The strength of the system lies in performing simultaneously two different biometric scans of the same person. This lowers the possibility of fraud dramatically compared to the classic approach. A reliable database was obtained using the proposed system. A neural network suited for the case was proposed and evaluated for validation of a user of the system. The achieved TPR goes up even to 99.5% by the EER threshold. It also proves that the proposed neural network architecture can be used for many different similar tasks. Traditionally, it would be advisable to design another set of methods for palm print and palm vein scans. With the use of CNNs, it is not needed. As a result a more reliable and error-prone biometric, user-friendly palm vein system boosted by UV scans was developed and validated.

## Figures and Tables

**Table 1:** Detailed database composition.

Number of	NIR Images	UV Images	Hands
Left hands	5080	5080	1030
Right hands	5080	5080	1030
Train group	3090	3090	1030
Validation group	1030	1030	1030
Test group	1030	1030	1030

**Table 2:** Comparison of hand position variability.

Database	X Position Variability [%]	Y Position Variability [%]
CASIA Palm Vein Database	3.77	4.15
Our database	7.80	8.38

**Table 3:** Comparison of methods.

Method	EER [%]	Size	Database
Kang et al. [28]	0.996	100 people	CASIA Palm Vein
Kang et al. [28]	3.112	105 people	author
Cancian et al. [27]	1.44	21 people	author
Khan et al. [45]	0.778	100 people	CASIA Palm Vein
Hao et al. [46]	0.72	100 people	CASIA Palm Vein
Hao et al. [46]	0.5	330 hands	author
Xuekui Yan et al. [47]	0.16	100 people	CASIA Palm Vein
Proposed method	0.5	1030 hands	author

## Abbreviations

The following abbreviations are used in this manuscript:

TPR	True Positive Rate
EER	Equal Error Rate
NIR	Near Infrared
UV	Ultraviolet
DNN	Deep Neural Network
SIFT	Scale-Invariant Feature Transform
FRR	False Rejection Rate
FAR	False Acceptance Rate
LBP	Local Binary Patterns
CCD	Charge Coupled Device
CNN	Convolutional Neural Network
PReLU	Parametric Rectified Linear Unit
ROI	Region of Interest
TNR	True Negative Rate
ROC	Receiver Operating Characteristic
FNR	False Negative Rate
FPR	False Positive Rate
LED	Light-emitting Diode
